# Ocular Biometric and Fundus Characteristics in High Myopia: Protocol for a Hospital-Based Cross-Sectional Study

**DOI:** 10.2196/96402

**Published:** 2026-11-03

**Authors:** Anshool Kumbhare, Pranaykumar Shinde

**Affiliations:** 1 Department of Ophthalmology, Jawaharlal Nehru Medical College, Datta Meghe Institute of Higher Education and Research, Sawangi (Meghe), Wardha, Wardha, Maharashtra, 442107, India, 91 9834497650

**Keywords:** high myopia, axial length, corneal thickness, keratometry, myopic maculopathy, META-PM classification

## Abstract

**Background:**

High myopia (spherical equivalent refractive error [SER] ≤–6.00 diopters) is a prominent global cause of visual impairment, but its structural alterations are inadequately documented at all severity levels in Indian tertiary hospitals.

**Objective:**

The aim of this study is to evaluate ocular biometric and fundus traits in high myopia and their relationship to the severity of myopia. The primary outcome is axial length measured using contact A-scan biometry. Meta-Analysis for Pathologic Myopia (META-PM) identified fundus changes, keratometry, central corneal thickness, and refractive astigmatism as secondary outcomes.

**Methods:**

This hospital-based cross-sectional observational study at Acharya Vinoba Bhave Rural Hospital, India, will enroll 160 participants aged 10 to 50 years with high myopia (SER ≤−6.00 diopters in ≥1 eye) consecutively over 2 years. Ophthalmic examination will include best-corrected visual acuity (converted to logarithm of the minimum angle of resolution [logMAR]), cycloplegic refraction, slit-lamp biomicroscopy, keratometry, pachymetry, A-scan biometry, and dilated fundus examination (META-PM). Statistical analysis will involve the Shapiro-Wilk test, Pearson/Spearman correlation, ANOVA/Kruskal-Wallis, chi-square, and generalized estimating equations using SPSS (version 23; IBM Corp).

**Results:**

The study received ethics approval on July 9, 2025. Enrollment has been ongoing since January 2026. Results are expected to be published in 2027 or 2028.

**Conclusions:**

This study will provide region-specific data and guide evidence-based high myopia management.

## Introduction

### Background

Myopia is a common vision problem worldwide (defined as spherical equivalent refractive error (SER) ≤−0.50 diopters [D]), but high myopia (SER ≤−6.00 D) is more serious [1]. It’s not just an optical inconvenience but a structural disease where progressive axial elongation raises the risk of vision-threatening concerns like myopic macular degeneration, glaucoma, retinal detachment, and cataracts [2,3]. Since risk increases exponentially with worsening myopia, distinguishing high myopia from low-to-moderate is clinically essential [1,3].

The prevalence of myopia has soared due to urbanization, intense schooling, and less outdoor time [4]. In Asia, up to 90% of school-aged children are myopic, and high myopia has a prevalence of up to 30% [5,6]. The prevalence of high myopia in 12-year-old children is 6.0%‐7.0% in Cambodia and New Delhi and 49.7%‐62% in China, Singapore, and Japan [7-10]. By 2050, approximately half of the world is anticipated to have myopia (10% high myopia), more than double compared to 2000 (and a fivefold increase for high myopia) [11].

High myopia diminishes quality of life beyond just blurry vision. The structural changes (elongation of the globe and progressive tissue thinning, corneal curvature alterations and fundus lesions like Fuchs spot, lacquer cracks, posterior staphyloma, and myopic crescent) are clinically recognized, yet how these changes vary across different levels of myopia severity remains poorly understood, particularly in cohorts from Central India [2,3,12].

### Prior Work

Research has consistently shown that axial length is the key driver of myopia severity and is a strong predictor of structural complications [1,2]. Myopia control studies have used atropine, orthokeratology, progressive addition lenses, and defocus incorporated multiple segment lenses and have consistently used axial length as the primary end point [13-17], yet most studies focused on low-to-moderate myopia, leaving high myopia cohorts, particularly in South Asian tertiary care, largely uncharacterized [14].

Corneal parameters have also drawn attention in high myopia research. High myopia alters central corneal thickness (CCT) and corneal curvature, affecting refractive surgery candidacy and intraocular pressure measurement [3]. Astigmatism frequently coexists with high myopia, yet its precise distribution across myopia severity remains unclear in Indian cohorts.

Fundus changes are a critical concern in high myopia. The spectrum of funduscopic findings ranges from early signs (myopic crescent and tigroid fundus) to advanced lesions (posterior staphyloma, lacquer cracks, and choroidal neovascularization), each carrying distinct prognostic implications [2,12]. The Meta-Analysis for Pathologic Myopia (META-PM) classification standardizes grading of these lesions and is widely recommended [18]. A systematic META-PM–based evaluation of fundus changes across myopia severity levels therefore has considerable clinical value in a tertiary care hospital setting.

### Study Objectives

The primary objective is to assess axial length in patients with high myopia and examine its relationship with the severity of myopia and associated pathological fundus changes. The secondary objectives are to assess myopic fundus changes using the META-PM classification; evaluate keratometry, CCT, and refractive astigmatism in relation to myopia severity; and determine the correlation between best-corrected visual acuity (BCVA) and axial length across myopia subgroups.

### Outcome Measures

The primary outcome of the study will be axial length measured using contact A-scan ultrasound biometry and its association with the severity of high myopia.

Secondary outcomes will include the distribution and severity of myopic fundus changes graded using the META-PM classification, including lesions (lacquer cracks, posterior staphyloma, Fuchs spot, and peripheral retinal degeneration). Additional secondary outcomes will include CCT, keratometric parameters (K1 and K2), refractive astigmatism, and the association of BCVA with axial length and refractive severity.

## Methods

### Study Design and Setting

This will be a hospital-based, cross-sectional, observational study that will be conducted at Acharya Vinoba Bhave Rural Hospital, Jawaharlal Nehru Medical College, Datta Meghe Institute of Higher Education and Research (Deemed to be University) (DMIHER [DU]), Maharashtra, India. The total duration of data collection is 2 years from the date of commencement (January 2026 to December 2027).

### Sample Size

The sample size calculation followed the standard formula for estimating a single proportion:

n=*Z*² × p(1 − p) / d²

Where *Z*=1.96 at a 95% confidence interval, *P*=.10 based on prior studies reporting pathological fundus change prevalence in high myopia, and d=0.05 is the acceptable margin of error [2,3]. The calculated sample size was 139 and was rounded up to 160 to account for potential attrition and nonresponse.

### Participant Selection

Patients presenting to the Department of Ophthalmology, Acharya Vinoba Bhave Rural Hospital, who fulfill the selection criteria, will be consecutively enrolled. The proposed screening, eligibility assessment, and recruitment process for participants is illustrated in Figure 1.

**Figure 1.**
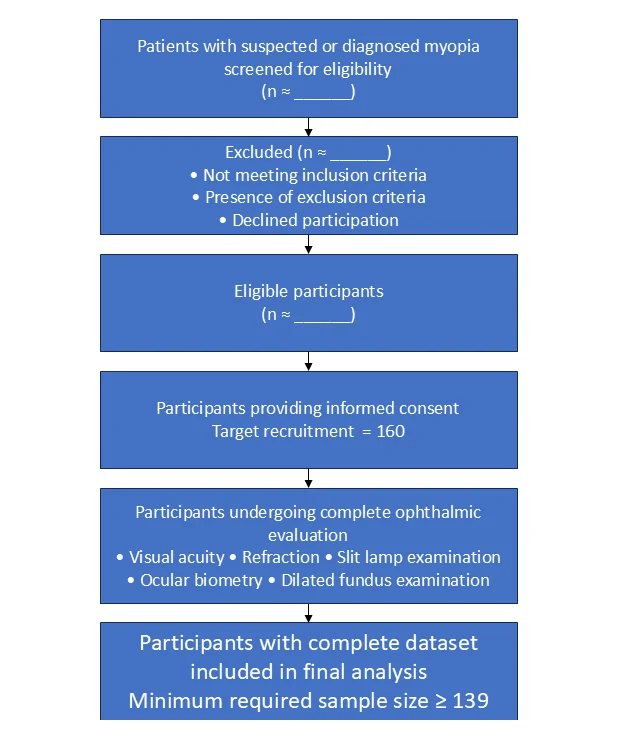
Proposed participant flow diagram showing screening, eligibility assessment, recruitment, and inclusion of participants in the cross-sectional study evaluating ocular biometric parameters and fundus characteristics in high myopia.

### Inclusion and Exclusion Criteria

Participants between 10 to 50 years of age with confirmed high myopia (defined as SER ≤−6.00 D in ≥1 eye on cycloplegic refraction) will be included. Both male and female participants willing to provide written informed consent (or written assent with parental/guardian consent for minors) will be enrolled. The upper age limit of 50 years was chosen to minimize confounding from age-related ocular changes including cataracts and age-related macular degeneration.

Patients with hypermetropia, active corneal lesions, significant lens opacities, active uveitis, previous intraocular or refractive surgery, or media opacities that preclude adequate fundus visualization will be excluded. Patients with systemic connective tissue disorders known to independently alter ocular biometric parameters (eg, Ehlers-Danlos syndrome, Marfan syndrome) will also be excluded. Individuals unwilling to participate or unable to comply with the examination protocol will not be enrolled.

### Ethical Considerations

Ethics approval was obtained from the institutional ethics committee of DMIHER (DU) (reference number DMIHER(DU)/IEC/2025/393, dated July 9, 2025). The study was prospectively registered with the Clinical Trials Registry – India (CTRI/2025/12/099253) on December 16, 2025. The study will adhere to the Declaration of Helsinki and the Indian Council of Medical Research National Ethical Guidelines (2017).

Written informed consent will be obtained from all participants prior to enrollment, with legal guardians providing consent for participants <18 years of age. Consent forms are available in English, Hindi, and Marathi (Multimedia Appendices 1 and 2). Participant confidentiality will be maintained; all data will be anonymized before analysis and publication. Participation will be voluntary, and withdrawal will not affect the standard clinical care.

### Data Collection

The principal investigator will perform all standardized ophthalmic evaluations and measurements to minimize interobserver variability. All instruments will be calibrated per manufacturer specifications (prior and during the study). Biometric measurements (axial length, CCT, and keratometry) will be averaged from 3 readings per eye. Pupillary dilation will use tropicamide 1% with or without phenylephrine 2.5%. Table 1 summarizes study variables and instruments. The assessments will be carried out for both eyes. A standardized proforma will be used to record all demographic, anthropometric, clinical, and biometric data (Multimedia Appendix 3).

**Table 1.** Study variables and measurement methods.

Variable	Measurement method	Instrument
Distance visual acuity	Snellen chart	Snellen chart at 6 m
Refractive error (SER)	Cycloplegic retinoscopy and subjective refraction	Retinoscope; autorefractometer
Axial length	Contact A-scan ultrasound biometry	A-scan biometer
Central corneal thickness	Contact ultrasound pachymetry	Ultrasound pachymeter
Corneal curvature (K1, K2)	Automated keratometry	Autokeratorefractometer
Astigmatism	Cycloplegic refraction	Retinoscope; autorefractometer
Anterior segment	Slit-lamp biomicroscopy	Slit lamp
Fundus (META-PM grading)	Dilated fundus examination	Direct and indirect ophthalmoscope

### Demographic and Anthropometric Data

Each participant’s age, sex, occupation, educational level, weight, height, and BMI will be noted.

### Visual Acuity

A Snellen chart will be used to measure BCVA for distance. Pinhole visual acuity will be recorded where needed. Snellen visual acuity will be rescaled to logarithm of the minimum angle of resolution (logMAR) units for statistical analysis.

### Refraction

Retinoscopy will be used for objective refraction, followed by subjective refinement. In children and adolescents, cycloplegia will be induced using cyclopentolate 1% (two drops administered 5 minutes apart), and refraction will be carried out 30 minutes after the last drop. Postmydriatic assessment will be carried out the following day. Automated refraction will be recorded using an autorefractometer for cross-reference. The SER for each eye will be determined by combining the sphere with half the cylindrical power.

### Slit-Lamp Biomicroscopy

Anterior segment evaluation will be performed to assess the cornea, anterior chamber, lens, and anterior vitreous for any relevant pathology.

### Fundus Examination

A posterior segment assessment will be performed using dilated direct and indirect ophthalmoscopy. Fundus findings will be documented and categorized according to the META-PM classification system, which grades pathologic myopia lesions into the following categories: no myopic retinopathy (Category 0), tessellated fundus only (Category 1), diffuse chorioretinal atrophy (Category 2), patchy chorioretinal atrophy (Category 3), and macular atrophy (Category 4) [18]. Additional findings (myopic crescent, lacquer cracks, Fuchs spot, peripheral retinal degeneration, and posterior staphyloma) will also be recorded separately. Archived images will be independently reviewed by a second ophthalmologist for consistent META-PM grading.

### Axial Length

Axial length will be measured using contact A-scan ultrasound biometry for each eye. Measurements will be analyzed across the following myopia severity subgroups based on SER: −6.00 D to −9.50 D, −10.00 D to −13.50 D, −14.00 D to −17.50 D, and ≤−18.00 D. These subgroup classifications were adapted from prior high-myopia stratification approaches used in pathological myopia and epidemiological studies [1,14,18].

### Corneal Pachymetry

CCT will be measured using a contact ultrasound pachymeter. Measurements will be classified as: ≤480 micrometers (very thin), 481 to 500 micrometers (thin), 501 to 520 micrometers (borderline), 521 to 540 micrometers (normal), 541 to 560 micrometers (above average), and >560 micrometers (thick).

### Keratometry

Corneal curvature (K1 and K2 readings) will be measured using automated keratometry. Mean keratometry values will be reported and correlated with the degree of myopia across the defined refractive subgroups.

### Astigmatism

Total refractive astigmatism will be recorded and classified as 0 D, ≤1.00 D, 1.00 to 2.00 D, and >2.00 D, and analyzed across each myopia severity subgroup.

### Bias and Missing Data Handling

Incomplete observations will be handled using complete-case analysis.

### Data Management and Statistical Analysis

All data will be entered and maintained in a Microsoft Excel (version 2018; Microsoft Corp) spreadsheet. Statistical analysis will be performed using SPSS (version 23; IBM Corp). Descriptive statistics (means, standard deviations, frequencies, and percentages) will be used to summarize demographic and clinical characteristics. Data normality will be assessed using the Shapiro-Wilk test. Pearson or Spearman correlation coefficients will be used to assess relationships between continuous variables (axial length SER and BCVA). Comparison of ocular parameters across myopia severity subgroups will be performed using a 1-way ANOVA for normally distributed data or the Kruskal-Wallis test for nonparametric data. Categorical variables, including fundus findings, will be analyzed using the chi-square test or Fisher exact test where expected cell counts are small. Generalized estimating equations will be applied to account for intereye correlation when both eyes are included in the analysis. Multivariable linear regression analysis will be performed after adjusting for potential confounders including age, sex, and BMI. A *P* value <.05 will be considered statistically significant for all analyses.

## Results

As of the submission of this protocol manuscript, the study has received full ethical clearance. Participant enrollment commenced in January 2026 and is currently ongoing. The target sample size is 160 participants. Results are expected to be available for analysis and publication by 2027 to 2028. The anticipated study timeline is presented in Table 2.

**Table 2.** Anticipated study timeline.

Phase	Activity	Period
Phase 1	Ethics approval and Clinical Trials Registry – India registration	Completed (July to December 2025)
Phase 2	Participant recruitment and data collection	January 2026 to December 2027
Phase 3	Data cleaning and statistical analysis	January 2028 to March 2028
Phase 4	Manuscript preparation and submission	April 2028 to June 2028

## Discussion

### Principal Findings (Anticipated)

This study protocol is designed to document the distribution of axial length, CCT, keratometry, astigmatism, and META-PM–graded fundus pathology across the graded spectrum of high myopia in a tertiary care cohort in Central India. We anticipate a strong positive correlation between axial length and myopia severity, with increasing frequency and severity of pathological fundus findings in higher myopia subgroups. Variation in CCT and keratometric values across subgroups will have direct implications for refractive surgery planning.

### Comparison With Prior Work

Prior reviews have consistently identified axial length as the key biomarker of myopia progression [13-17]. A recent review noted the scarcity of dedicated high-myopia data despite disproportionate complications risk [14]. Our study addresses this gap by focusing solely on high myopia and recording extensive structural and biometric parameters.

While most prior research with large sample sizes has been conducted in East Asian populations, limited data exist that specifically characterize the comprehensive panel of ocular parameters, including axial length, corneal findings, and fundus changes, in a high myopia cohort from Central India [5,6,9,10]. Differences in ethnic background, behavioral patterns, and environmental exposures may produce findings that diverge from East Asian cohorts, and our study is positioned to contribute these region-specific data [19,20].

### Clinical Implications

The anticipated findings of this study may improve risk stratification and clinical monitoring strategies in highly myopic patients attending tertiary care centers. Identification of ocular biometric markers associated with pathological fundus changes may support earlier surveillance and intervention in patients at higher risk of visual morbidity. The findings may also assist clinicians in refractive surgery evaluation and long-term management planning in highly myopic populations.

### Strengths and Limitations

This study has several notable strengths. This will be the first prospectively registered, ethics-approved cross-sectional study in Central India to document a comprehensive panel of ocular biometric and structural parameters (axial length, CCT, keratometry, astigmatism, and META-PM–graded fundus findings) specifically in a high myopia cohort. The validated META-PM classification system will ensure reproducibility and comparability, while generalized estimating equations will account for intereye correlation, strengthening analytical rigor.

Several limitations are acknowledged. The cross-sectional design will preclude causality and temporal progression of ocular changes. Hospital-based recruitment may introduce selection bias, as a tertiary referral center may see more severe disease than the community populations. The absence of an emmetropic control group will limit direct comparison with normative parameters. Single center conduct may affect generalizability across India’s diverse population. Additionally, contact ultrasound biometry may have greater measurement variability than optical biometry. These limitations will be discussed in detail in the final publication of results.

### Future Directions

Future longitudinal studies using optical biometry and multimodal retinal imaging may clarify temporal progression of structural ocular changes in high myopia. Future multicenter studies with larger, diverse populations may improve generalizability and enable region-specific screening for pathological myopia.

### Dissemination Plan

Study findings will be disseminated through peer-reviewed journal publications, conference presentations, and institutional academic forums. Deidentified aggregate data may contribute to future regional epidemiological research on high myopia and pathological myopia.

### Conclusions

This protocol presents a comprehensive, ethics-approved, and prospectively registered cross-sectional study evaluating ocular parameters in patients with high myopia at a tertiary care hospital in Central India. The study is designed to generate clinically relevant data on fundus changes, axial length, keratometry, CCT, and astigmatism across the spectrum of high myopia. Findings from this study are expected to inform evidence-based approaches to the monitoring and management of high myopia in the Indian population and contribute to the growing global body of literature on this significant public health issue.

## Supplementary material

10.2196/96402 Multimedia Appendix 1 Patient information sheet.

10.2196/96402 Multimedia Appendix 2 Patient consent form.

10.2196/96402 Multimedia Appendix 3 Proforma for data collection.

## Abbreviations

BCVA: best-corrected visual acuity
CCT: central corneal thickness
D: diopters
DMIHER (DU): Datta Meghe Institute of Higher Education and Research (Deemed to be University)
logMAR: logarithm of the minimum angle of resolution
META-PM: Meta-Analysis for Pathologic Myopia
SER: spherical equivalent refractive error
