# Supplementary material for: Ocular Biometric and Fundus Characteristics in High Myopia: Protocol for a Hospital-Based Cross-Sectional Study
Source: JMIR Res Protoc. 2026 Nov 3;15:e96402. doi: 10.2196/96402 (PMC13730406; doi:10.2196/96402)
Supplement: Supplementary file 1 [file resprot-v15-e96402-s001.docx]

**PATIENT INFORMATION SHEET (ENGLISH)**

Title of Study:
Ocular Biometric and Fundus Characteristics in High Myopia: A Hospital-Based Cross-Sectional Study

Principal Investigator:
Dr. Anshool Anil Kumbhare
Department of Ophthalmology
Jawaharlal Nehru Medical College & Acharya Vinoba Bhave Rural Hospital
Datta Meghe Institute of Higher Education and Research (DMIHER), Wardha

1. Introduction

You are being invited to participate in a research study conducted by the Department of Ophthalmology at AVBRH, Wardha. Before you decide whether to participate, it is important for you to understand why the research is being done and what it will involve.

Please read the following information carefully. If you have any questions, feel free to ask the investigator.

2. Purpose of the Study

This study aims to evaluate certain eye measurements and retinal findings in patients with high myopia (high power minus glasses). The information obtained from this study may help doctors better understand changes that occur in highly myopic eyes and improve patient care in the future.

3. Why Have I Been Invited?

You are being invited because you have high myopia (power of −6.00 diopters or more) or symptoms related to it. Around 140 participants will take part in this study.

4. Procedures Involved

If you agree to participate, the following eye examinations will be performed:

- Measurement of visual acuity (vision testing)
- Refraction test to determine the power of glasses
- Slit-lamp examination of the front part of the eye
- Measurement of axial length (length of the eyeball)
- Measurement of corneal thickness
- Keratometry to measure corneal curvature
- Dilated fundus examination to examine the retina

These procedures are routine ophthalmic tests and will take approximately 30–45 minutes.

5. Possible Risks or Discomfort

The procedures involved in this study are non-invasive and routinely performed in eye clinics.

You may experience:

- Temporary blurred vision after dilating eye drops
- Mild discomfort during some tests

These effects usually resolve within a few hours.

6. Benefits of Participation

You may not receive direct benefits from participating in this study. However, the examinations may help identify eye problems early and contribute to better understanding of high myopia.

7. Confidentiality

All information collected during this study will be kept strictly confidential.

- Your identity will not be disclosed in any reports or publications.
- Data will be stored securely and accessed only by the research team.

8. Voluntary Participation

Your participation in this study is completely voluntary.

- You may refuse to participate.
- You may withdraw from the study at any time without affecting your medical care.

9. Compensation

No financial compensation will be provided for participation in this study. All routine medical care will continue as usual.

10. Contact Information

If you have any questions regarding the study, please contact:

Principal Investigator:
Dr. Anshool Anil Kumbhare
Department of Ophthalmology
JNMC & AVBRH, Wardha

If you have concerns about your rights as a participant, you may contact the Institutional Ethics Committee, DMIHER.

**रोगी सूचना पत्र (HINDI)**

अध्ययन का शीर्षक:
उच्च मायोपिया में नेत्रीय जैवमितीय एवं फंडस विशेषताओं का अध्ययन

मुख्य अन्वेषक:
डॉ. अंशूल अनिल कुम्भारे
नेत्र रोग विभाग
जेएनएमसी एवं एवीबीआरएच, डीएमआईएचईआर, वर्धा

1. परिचय

आपको इस शोध अध्ययन में भाग लेने के लिए आमंत्रित किया जा रहा है। इस अध्ययन का उद्देश्य उच्च मायोपिया वाले रोगियों की आंखों में होने वाले परिवर्तनों का अध्ययन करना है।

कृपया नीचे दी गई जानकारी ध्यान से पढ़ें। यदि आपको कोई प्रश्न हो तो आप शोधकर्ता से पूछ सकते हैं।

2. अध्ययन का उद्देश्य

इस अध्ययन का उद्देश्य उच्च मायोपिया वाले रोगियों में आंख की संरचना और रेटिना में होने वाले परिवर्तनों का मूल्यांकन करना है।

3. आपको क्यों चुना गया है?

आपको इसलिए चुना गया है क्योंकि आपको उच्च मायोपिया (−6.00 डायोप्टर या उससे अधिक) है।

इस अध्ययन में लगभग 140 प्रतिभागी शामिल होंगे।

4. अध्ययन में क्या किया जाएगा?

यदि आप भाग लेने के लिए सहमत होते हैं, तो निम्नलिखित परीक्षण किए जाएंगे:

- दृष्टि परीक्षण
- चश्मे का नंबर जांचना
- स्लिट-लैम्प जांच
- आंख की लंबाई मापना
- कॉर्निया की मोटाई मापना
- कॉर्निया की वक्रता मापना
- पुतली को फैलाकर रेटिना की जांच

इन सभी परीक्षणों में लगभग 30–45 मिनट लगेंगे।

5. संभावित जोखिम

इस अध्ययन में उपयोग किए जाने वाले परीक्षण सामान्य नेत्र जांच का हिस्सा हैं।

संभावित असुविधाएँ:

- आंखों में डाले गए ड्रॉप्स के कारण कुछ समय के लिए धुंधला दिखना
- हल्की असुविधा

ये प्रभाव सामान्यतः कुछ घंटों में समाप्त हो जाते हैं।

6. लाभ

इस अध्ययन से आपको सीधे लाभ नहीं मिल सकता है, लेकिन यह भविष्य में रोगियों के बेहतर उपचार में सहायक हो सकता है।

7. गोपनीयता

आपकी सभी जानकारी गोपनीय रखी जाएगी।

आपका नाम किसी भी रिपोर्ट या प्रकाशन में प्रकट नहीं किया जाएगा।

8. स्वैच्छिक भागीदारी

इस अध्ययन में भाग लेना पूरी तरह स्वैच्छिक है।

आप किसी भी समय बिना कारण बताए अध्ययन से बाहर हो सकते हैं।

9. मुआवज़ा
इस अध्ययन में भाग लेने के लिए कोई वित्तीय मुआवज़ा प्रदान नहीं किया जाएगा। सभी नियमित चिकित्सा सेवाएँ सामान्य रूप से जारी रहेंगी।

10. संपर्क जानकारी

यदि आपको अध्ययन के बारे में कोई प्रश्न है, तो आप संपर्क कर सकते हैं:

डॉ. अंशूल अनिल कुम्भारे
नेत्र रोग विभाग
जेएनएमसी एवं एवीबीआरएच, वर्धा

**रुग्ण माहिती पत्रक (MARATHI)**

अभ्यासाचे शीर्षक:
उच्च मायोपियामधील डोळ्यांच्या जैवमितीय आणि फंडस वैशिष्ट्यांचा अभ्यास

मुख्य संशोधक:
डॉ. अंशूल अनिल कुंभारे
नेत्ररोग विभाग
JNMC आणि AVBRH, DMIHER, वर्धा

1. प्रस्तावना

आपल्याला या संशोधन अभ्यासामध्ये सहभागी होण्यासाठी आमंत्रित केले आहे. या अभ्यासाचा उद्देश उच्च मायोपिया असलेल्या रुग्णांमध्ये डोळ्यांमध्ये होणारे बदल समजून घेणे आहे.

2. अभ्यासाचा उद्देश

या अभ्यासामध्ये उच्च मायोपिया असलेल्या रुग्णांमध्ये डोळ्यांच्या संरचनेतील आणि रेटिनामधील बदलांचा अभ्यास केला जाईल.

3. आपण का निवडले गेले आहात?

आपल्याला उच्च मायोपिया (−6.00 डायॉप्टर किंवा त्यापेक्षा जास्त) असल्यामुळे या अभ्यासासाठी निवडले आहे.

या अभ्यासात सुमारे 140 रुग्ण सहभागी होतील.

4. अभ्यासामध्ये काय केले जाईल?

आपण सहभागी होण्यास सहमत असल्यास खालील तपासण्या केल्या जातील:

- दृष्टी तपासणी
- चष्म्याचा नंबर तपासणी
- स्लिट लॅम्प तपासणी
- डोळ्याची लांबी मोजणे
- कॉर्नियाची जाडी मोजणे
- कॉर्नियाची वक्रता तपासणे
- डोळ्यात ड्रॉप टाकून रेटिनाची तपासणी

या सर्व तपासण्यांना सुमारे 30–45 मिनिटे लागतील.

5. संभाव्य धोके

या तपासण्या नियमित नेत्र तपासण्यांचा भाग आहेत.

काही वेळा:

- डोळ्यांत टाकलेल्या ड्रॉप्समुळे तात्पुरते धूसर दिसू शकते
- थोडी अस्वस्थता जाणवू शकते

हे परिणाम काही तासांत कमी होतात.

6. लाभ

या अभ्यासातून आपल्याला थेट फायदा होणार नाही, परंतु भविष्यात रुग्णांच्या उपचारात सुधारणा होण्यास मदत होईल.

7. गोपनीयता

आपली सर्व माहिती पूर्णपणे गोपनीय ठेवली जाईल.

आपले नाव कोणत्याही अहवाल किंवा प्रकाशनात वापरले जाणार नाही.

8. स्वेच्छा सहभाग

या अभ्यासामध्ये सहभागी होणे पूर्णपणे स्वेच्छेचे आहे.

आपण कोणत्याही वेळी अभ्यासातून बाहेर पडू शकता.

9. भरपाई
या अभ्यासामध्ये सहभागी होण्यासाठी कोणतीही आर्थिक भरपाई दिली जाणार नाही. सर्व नियमित वैद्यकीय सेवा नेहमीप्रमाणे सुरू राहतील.

10. संपर्क

अभ्यासाबाबत काही प्रश्न असल्यास कृपया संपर्क साधा:

डॉ. अंशूल अनिल कुंभारे
नेत्ररोग विभाग
JNMC आणि AVBRH, वर्धा
