# Supplementary material for: Ocular Biometric and Fundus Characteristics in High Myopia: Protocol for a Hospital-Based Cross-Sectional Study
Source: JMIR Res Protoc. 2026 Nov 3;15:e96402. doi: 10.2196/96402 (PMC13730406; doi:10.2196/96402)
Supplement: Supplementary file 2 [file resprot-v15-e96402-s002.docx]

**INFORMED CONSENT FORM (ENGLISH)**

Title of Study:
Ocular Biometric and Fundus Characteristics in High Myopia: A Hospital-Based Cross-Sectional Study

Principal Investigator:
Dr. Anshool Anil Kumbhare
Department of Ophthalmology
Jawaharlal Nehru Medical College & AVBRH
Datta Meghe Institute of Higher Education and Research (DMIHER), Wardha

Participant Consent

I confirm that:

- I have read the Patient Information Sheet, or it has been explained to me in a language that I understand.
- I have had the opportunity to ask questions about the study and all my questions have been answered satisfactorily.
- I understand the purpose, procedures, possible risks, and benefits of the study.
- I understand that my participation in this study is voluntary.
- I understand that I may withdraw from the study at any time without affecting my medical care.
- I understand that my identity and personal information will be kept confidential.
- I agree that the data collected during the study may be used for research and scientific publication, without revealing my identity.

I voluntarily agree to participate in this research study.

Name of Participant: ___________________________

Age/Gender: ___________________________

Signature/Thumb Impression of Participant: ___________________________

Date: ___________________________

If the participant is illiterate, the information has been read out and explained in a language understood by them.

Name of Witness: ___________________________

Signature of Witness: ___________________________

Date: ___________________________

Investigator Declaration

I confirm that I have explained the nature and purpose of the study, procedures involved, potential risks and benefits, and the rights of the participant in a language they understand.

Name of Investigator: ___________________________

Signature of Investigator: ___________________________

Date: ___________________________

**सूचित सहमति प्रपत्र (HINDI)**

अध्ययन का शीर्षक:
उच्च मायोपिया में नेत्रीय जैवमितीय एवं फंडस विशेषताओं का अध्ययन

मुख्य अन्वेषक:
डॉ. अंशूल अनिल कुम्भारे
नेत्र रोग विभाग
जेएनएमसी एवं एवीबीआरएच, डीएमआईएचईआर, वर्धा

प्रतिभागी की सहमति

मैं यह पुष्टि करता/करती हूँ कि:

- मैंने रोगी सूचना पत्र पढ़ लिया है या मुझे समझाई गई है।
- मुझे इस अध्ययन के बारे में प्रश्न पूछने का अवसर दिया गया और मेरे सभी प्रश्नों के उत्तर दिए गए।
- मुझे अध्ययन का उद्देश्य, प्रक्रिया, संभावित जोखिम और लाभ समझ में आ गए हैं।
- मुझे बताया गया है कि इस अध्ययन में भाग लेना पूर्णतः स्वैच्छिक है।
- मैं किसी भी समय बिना किसी कारण बताए अध्ययन से बाहर हो सकता/सकती हूँ।
- मेरी व्यक्तिगत जानकारी गोपनीय रखी जाएगी।
- अध्ययन के परिणामों का उपयोग वैज्ञानिक प्रकाशनों में किया जा सकता है, लेकिन मेरी पहचान उजागर नहीं की जाएगी।

मैं स्वेच्छा से इस अध्ययन में भाग लेने के लिए सहमत हूँ।

प्रतिभागी का नाम: ___________________________

आयु/लिंग: ___________________________

प्रतिभागी के हस्ताक्षर/अंगूठा निशान: ___________________________

तारीख: ___________________________

यदि प्रतिभागी पढ़-लिख नहीं सकता, तो जानकारी उसे उसकी समझ की भाषा में पढ़कर सुनाई गई।

गवाह का नाम: ___________________________

गवाह के हस्ताक्षर: ___________________________

तारीख: ___________________________

अन्वेषक की घोषणा

मैं यह पुष्टि करता/करती हूँ कि मैंने प्रतिभागी को अध्ययन का उद्देश्य, प्रक्रिया, जोखिम और लाभ उनकी समझ की भाषा में समझा दिए हैं।

अन्वेषक का नाम: ___________________________

अन्वेषक के हस्ताक्षर: ___________________________

तारीख: ___________________________

**सूचित संमतीपत्र (MARATHI)**

अभ्यासाचे शीर्षक:
उच्च मायोपियामधील डोळ्यांच्या जैवमितीय आणि फंडस वैशिष्ट्यांचा अभ्यास

मुख्य संशोधक:
डॉ. अंशूल अनिल कुंभारे
नेत्ररोग विभाग
JNMC आणि AVBRH
DMIHER, वर्धा

सहभागीची संमती

मी खालील बाबी समजून घेतल्या आहेत:

- मला रुग्ण माहिती पत्रक वाचून दाखवले गेले आहे किंवा समजावून सांगितले आहे.
- मला अभ्यासाबद्दल प्रश्न विचारण्याची संधी मिळाली आणि माझ्या सर्व प्रश्नांची उत्तरे मिळाली.
- मला अभ्यासाचा उद्देश, प्रक्रिया, संभाव्य धोके आणि फायदे समजले आहेत.
- या अभ्यासामध्ये सहभागी होणे पूर्णपणे स्वेच्छेचे आहे.
- मी कोणत्याही वेळी कोणतेही कारण न देता अभ्यासातून बाहेर पडू शकतो/शकते.
- माझी वैयक्तिक माहिती गोपनीय ठेवली जाईल.
- अभ्यासातील माहिती वैज्ञानिक प्रकाशनासाठी वापरली जाऊ शकते, परंतु माझी ओळख उघड केली जाणार नाही.

मी स्वेच्छेने या संशोधन अभ्यासात सहभागी होण्यास सहमत आहे.

सहभागीचे नाव: ___________________________

वय/लिंग: ___________________________

सहभागीची सही/अंगठा ठसा: ___________________________

दिनांक: ___________________________

जर सहभागी निरक्षर असेल, तर माहिती त्याला/तिला समजेल अशा भाषेत वाचून सांगितली आहे.

साक्षीदाराचे नाव: ___________________________

साक्षीदाराची सही: ___________________________

दिनांक: ___________________________

संशोधकाची घोषणा

मी सहभागीला अभ्यासाचा उद्देश, प्रक्रिया, संभाव्य धोके आणि फायदे त्यांच्या समजेल अशा भाषेत समजावून सांगितले आहेत.

संशोधकाचे नाव: ___________________________

संशोधकाची सही: ___________________________

दिनांक: ___________________________
