# Supplementary material for: Ocular Biometric and Fundus Characteristics in High Myopia: Protocol for a Hospital-Based Cross-Sectional Study
Source: JMIR Res Protoc. 2026 Nov 3;15:e96402. doi: 10.2196/96402 (PMC13730406; doi:10.2196/96402)
Supplement: Supplementary file 3 [file resprot-v15-e96402-s003.docx]

**DATA COLLECTION PROFORMA (CRF)**

**Study Title:**
Ocular Biometric and Fundus Characteristics in High Myopia: A Hospital-Based Cross-Sectional Study

**Study Site:** Department of Ophthalmology, JNMC & AVBRH, DMIHER, Wardha

**Participant Study ID:** ________
**Date of Enrollment:** ________
**Examiner Name:** ________

**1. PARTICIPANT IDENTIFICATION**

| **Variable** | **Entry** |
| --- | --- |
| Hospital Registration Number |  |
| Study ID |  |
| Date of Visit |  |

**2. SOCIODEMOGRAPHIC VARIABLES (STROBE: Descriptive Data)**

| **Variable** | **Entry** |
| --- | --- |
| Age (years) |  |
| Sex | ☐ Male ☐ Female ☐ Other |
| Residence | ☐ Rural ☐ Urban |
| Occupation |  |
| Education Level | ☐ Illiterate ☐ Primary ☐ Secondary ☐ Graduate |

**3. ELIGIBILITY CRITERIA**

**Inclusion Criteria**

☐ Age ≥ 18 years
☐ High Myopia (≤ −6.00 D spherical equivalent)

**Exclusion Criteria**

☐ Previous retinal surgery
☐ Ocular trauma
☐ Media opacity preventing fundus examination
☐ Other retinal pathology unrelated to myopia

Eligible: ☐ Yes ☐ No

**4. MEDICAL HISTORY (STROBE: Potential Confounders)**

| **Variable** | **Entry** |
| --- | --- |
| Age of onset of myopia |  |
| Duration of myopia (years) |  |
| Family history of myopia | ☐ Yes ☐ No |
| History of spectacle use | ☐ Yes ☐ No |
| Contact lens use | ☐ Yes ☐ No |
| Systemic diseases | ☐ Diabetes ☐ Hypertension ☐ None |
| Medication history |  |

**5. VISUAL ACUITY**

| **Parameter** | **Right Eye (OD)** | **Left Eye (OS)** |
| --- | --- | --- |
| Unaided Visual Acuity |  |  |
| Best Corrected Visual Acuity |  |  |
| Near Vision |  |  |

**6. REFRACTIVE STATUS (STROBE: Exposure Variable)**

| **Parameter** | **Right Eye** | **Left Eye** |
| --- | --- | --- |
| Sphere (D) |  |  |
| Cylinder (D) |  |  |
| Axis |  |  |
| Spherical Equivalent |  |  |

**7. ANTERIOR SEGMENT EXAMINATION**

(Slit Lamp)

| **Finding** | **OD** | **OS** |
| --- | --- | --- |
| Lids |  |  |
| Conjunctiva |  |  |
| Cornea |  |  |
| Anterior Chamber |  |  |
| Iris |  |  |
| Lens |  |  |

**8. OCULAR BIOMETRIC PARAMETERS (Primary Study Variables)**

| **Parameter** | **OD** | **OS** |
| --- | --- | --- |
| Axial Length (mm) |  |  |
| Anterior Chamber Depth (mm) |  |  |
| Lens Thickness (mm) |  |  |
| Keratometry K1 |  |  |
| Keratometry K2 |  |  |
| Central Corneal Thickness (µm) |  |  |

**9. INTRAOCULAR PRESSURE**

| **Parameter** | **OD** | **OS** |
| --- | --- | --- |
| IOP (mmHg) |  |  |
| Measurement Method |  |  |

**10. POSTERIOR SEGMENT / FUNDUS FINDINGS (Outcome Variables)**

| **Finding** | **OD** | **OS** |
| --- | --- | --- |
| Optic Disc Appearance |  |  |
| Cup Disc Ratio |  |  |
| Peripapillary Atrophy | ☐ Yes ☐ No | ☐ Yes ☐ No |
| Tessellated Fundus | ☐ Yes ☐ No | ☐ Yes ☐ No |
| Posterior Staphyloma | ☐ Yes ☐ No | ☐ Yes ☐ No |
| Lacquer Cracks | ☐ Yes ☐ No | ☐ Yes ☐ No |
| Chorioretinal Atrophy | ☐ Yes ☐ No | ☐ Yes ☐ No |
| Myopic Maculopathy Grade |  |  |
| Retinal Detachment | ☐ Yes ☐ No | ☐ Yes ☐ No |
| Other Findings |  |  |

**11. IMAGING FINDINGS (IF AVAILABLE)**

| **Investigation** | **OD** | **OS** |
| --- | --- | --- |
| OCT Findings |  |  |
| Fundus Photography |  |  |
| B-Scan |  |  |

**12. FINAL CLINICAL CLASSIFICATION**

| **Parameter** | **Entry** |
| --- | --- |
| Degree of Myopia | ☐ Moderate ☐ High |
| Pathologic Myopia | ☐ Yes ☐ No |
| META-PM Classification |  |

**13. MANAGEMENT / ADVICE**

| **Variable** | **Entry** |
| --- | --- |
| Spectacle prescription |  |
| Referral required | ☐ Yes ☐ No |
| Follow-up advised |  |

**14. DATA QUALITY CONTROL (STROBE REQUIREMENT)**

| **Variable** | **Entry** |
| --- | --- |
| Data checked by |  |
| Date |  |
| Signature |  |
